# COVID-19: A Source of Stress and Depression Among University Students and Poor Academic Performance

**DOI:** 10.3389/fpubh.2022.898556

**Published:** 2022-04-25

**Authors:** Zuopeng Jiang, Xuhong Jia, Ran Tao, Hazar Dördüncü

**Affiliations:** ^1^International Business School, Qingdao Huanghai University, Qingdao, China; ^2^Qingdao Municipal Center for Disease Control and Prevention, Qingdao, China; ^3^Faculty of Economics, Administrative and Social Sciences, Department of International Trade and Logistics, Nisantasi University, Istanbul, Turkey

**Keywords:** academic stress, family stress, student learning, academic performance, COVID-19

## Abstract

Current research examines how COVID-19 has impacted the daily life of students, specifically personal and academic aspects. The authors investigated the role of academic and family stress caused by COVID-19 on students' depression levels and the subsequent impact on their academic performance based on Lazarus' cognitive appraisal theory of stress. The non-probability convenience sampling technique has been used to collect data from undergraduate and postgraduate students using a modified questionnaire with a five-point Likert scale. This study used structural equation modeling to examine the link between stress, depression, and academic performance during COVID-19. It was confirmed that educational and family stress significantly leads to depression among students, negatively affecting their academic performance and learning outcomes. This research provides valuable information to parents, educators, and other stakeholders concerned about their children's education and performance.

## Introduction

The outbreak of coronavirus diseases (COVID-19) has had a significant impact on the lives of people worldwide (1), particularly since the World Health Organization (WHO) declared a global pandemic (2) in the second week of March 2020. As a result, many countries implemented various anti-epidemic measures, including limiting foreign nationals' travel (1), closing public spaces, and shutting down the entire transit system (2) to contain the spread of highly contagious infections from human to human. The education sector is not immune to the fact that the conventional educational system is no longer effective, and academic institutions worldwide are exploring online education alternatives (3). This is because all educational institutions worldwide have been closed due to lockdowns, and the students cannot meet their teachers in person. COVID-19 is one of the most stressful pandemics that humanity has ever experienced. It has drastically disrupted people's daily routines and negatively affects their physical and mental (1). The impact of such incidents on students' psychological health is rarely investigated, and in Pakistan, inadequate information exists on it (4). Xiong et al. (2) published pioneering research on the general population in Italy, Turkey, Iran, Spain, Nepal, and the United States. The pandemic, it was declared, affects young and older people differently (5). As a result, it was determined that sociodemographic predictors of mental distress in students and their level of satisfaction with the continuous blended learning mode were necessary.

On February 26, 2020, the first case of COVID-19 was reported in Pakistan (6). As of March 20th, 2021, over 623,135 confirmed cases. To curb and contain the virus, the government instructed educational institutions to shut down their operations on March 13th, 2020. Following this, on March 23rd, 2020, a 14 days lockdown was enforced in which all unnecessary activities in daily life were prohibited (7). As with China and Italy, the nationwide smart lockdown began on March 27, 2020, later phased out (8). The goal of the activity restrictions was to save lives by preventing viral transmission, reducing its incidence, and alleviating the burden on the medical care system (9). School closures in response to pandemics such as the flu have remained a successful strategy for lowering virus transmission rates and flattening disease incidence peaks (9, 10). This strategy appeared to be quite effective because it minimized student-student contact and protected students from infection (11). On the other hand, it impacted students and the general public (9, 12). Wang et al. reported that restricted routine activities and self-isolation had a significant psychological impact on people (13). Recently, it was discovered that the COVID-19 pandemic is causing psychological distress in some people (14, 15).

One of the essential pillars of any nation's development is its higher education system (16). HEIs' success is mainly dependent on the success of their students, who are the primary stakeholders (17). Having the necessary skills and abilities to compete in today's rapidly changing industrial environment is essential for students to succeed (18). In today's intensely competitive academic climate, various factors play a significant role in how well students perform academically (19, 20). According to Aafreen, Priya, and Gayathri (17), academic life is stressful for students because of the constant pressure from various sources. In numerous countries, university and school closures have impacted young people's mental health, increasing anxiety and loneliness (21). Based on findings from previous studies conducted during pandemics, the WHO recognized that imposing measures such as social isolation may increase individuals' anxiety, stress, and anger (22, 23). Stress is frequently felt due to a threat to psychological, intellectual, or somatic wellbeing (24, 25). Any form of change that creates emotional, physical, or psychological distress is called stress. Sometimes, it also promotes deviant behavior (23). It is an individual body's reaction to anything that demands attention or action. To some extent, everyone experiences stress. Individual morale suffers greatly when they are subjected to much stress (23). It manifests when a person cannot control their inner and outer emotions. Stress can harm an individual's mental health if it persists for an extended period or reaches a certain level (26). Suicidal thoughts and unhappiness are common symptoms of depression, affecting people worldwide (27, 28).

Similarly, depression harms one's energy, ability to focus, and ability to make career decisions (29). To build an educated society, students are essential. When students are depressed, their academic performance suffers, significantly impacting their lives. Possible reasons are family problems, new lifestyles in colleges and universities, and poor academic grades. Stress and academic pressure can also be significant factors in developing depression (30). Home quarantine, a lack of physical activity, uncertainty about the pandemic's trajectory, a lack of information, and fear of contracting COVID-19 were identified as risk factors for poor mental health among university students in Bangladesh (31). Moreover, fear of infection and a perceived high risk of infection were identified as factors affecting the mental health of university students in China (23, 31).

Multiple studies have investigated and confirmed that COVID-19 has increased stress and depression level in society. Some researchers investigated it from an organizational perspective, while some studied it from a family relationship perspective. However, the literature review indicates that rare attention is paid to this phenomenon from students' academic and family stress perspectives. Lazarus's theory, which focuses on a person's relationship to their environment, is used in this study to examine the impact of stress on a student's level of depression in the current pandemic period. It is also discussed how stress and depression impact students' academic performance. There are preliminary studies that examine the impact of stress on students' depression levels and academic performance during the COVID-19 pandemic, particularly in Pakistan, where the study was conducted (32). Apart from that, this study is unique. It examines the relationship between the variables listed using a multivariate statistical technique followed by structural equation modeling (SEM) and examines the stress by incorporating family and academic aspects.

## Theory and Literature

In 1966, a psychologist named Richard Lazarus published Psychological Stress and Coping, which pioneered the concept of cognitive appraisal theory (33). Appraisal and coping are central concepts in any theory of psychological stress, according to this theory (34), and there is a strong connection between the two. The theory holds that stress is caused by a discrepancy between the demands placed on individuals and their capacity to cope with those demands (35). As a result of the recent adaptation, stress is not defined as a specific cause of incitement or as an individual's psychological, behavioral, or subjective reaction. Instead, it is viewed as a relationship between a person and their surrounding circumstances (36). People see the environment as essential to their wellbeing, and they try to deal with the overwhelming demands and challenges that come with living in modern society (37).

The cognitive appraisal model is predicated on the notion that one's expectations about the significance and outcome of an event, encounter, or function affect stress and other emotional processes (35). This explains why different people's reactions to the same environment elicit different levels of intensity, duration, and quality of emotion (15). Primary and secondary appraisals can be influenced by a wide range of factors (such as goals and values), and specific patterns can lead to different types of stress (38). Stress can cause a variety of mental and physical reactions in other people. According to Semedo et al. (39), individuals may experience stress due to the external environment or subjective feelings, resulting in mental health issues like anxiety and depression. Stress can have adverse effects on one's health (40). Due to the high-stress levels, students' learning outcomes have been adversely affected (26). Stress can be dealt with in a variety of ways. Identifying the root causes of stress can lead to terms like family stress and academic stress, among others.

### Academic Stress and Students' Depression Levels

Adults' mental health is generally thought to improve, and depression disorder decreases between 18 and 25 years. On the other hand, high rates of depression are becoming more common (41), and many university students are testing above the clinical cut-off points for severe depression in this particular screen (42). According to Aafreen, Priya, and Gayathri (43), 30 percent of high school students suffer from depression in various ways. As a result, many recent high school graduates face an increased risk of developing depression upon entering college (44). Students' stress levels rise as they progress through the educational system. This is due in part to increasingly difficult coursework, tighter deadlines on assignments, and issues with finding housing for students who have relocated from other cities. Students' university experiences may also play a role in developing depressive symptoms. Subjective and objective experiences are closely linked to depressive disorders. The stress that comes with being a student at a university contributes to the wide range of depressive experiences that students have.

In a survey of students from Canadian universities, 42.3% of respondents said they had experienced severe anxiety and stress (45). Furthermore, 58.1% of students said academic projects are too complex to handle. The majority of Germans, Bulgarians, and Poles view assignments as a burden that cannot be compared to other concerns in life (41). Multiple studies on university students' stress have found a relationship between their educational needs and demands and depression disorder and apparent anxiety. Lörz et al. (46) found that even after controlling for 13 different risk factors for depression in a cross-sectional study of 900 Canadian university students, stress experienced as a result of academic workload was associated with high levels of negative symptoms (e.g., demographic features, abusive past, intellectual way, and personality, currently experienced stressful trials in life, societal support). Few studies have shown that students who complain about their academic workload or label it “traumatic” are more likely to suffer from depressive disorders (30).

**Figure 1:**
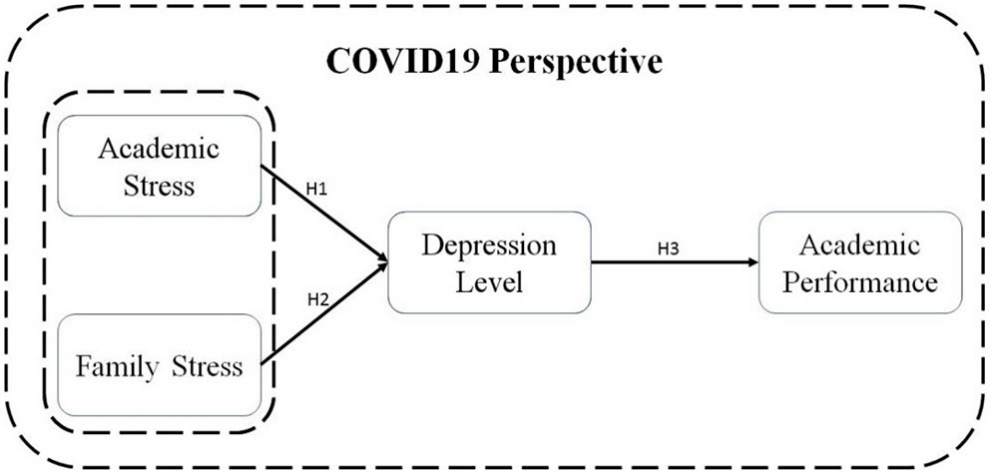
Conceptual model.

In the current pandemic period, students are advised to consider all of the potential sources of stress before enrolling in college. The pandemic has created a sense of fear among students in ety. Thus, it is claimed that academic pressure created during the pandemic situation causes depression among students (see Figure 1). For this reason, the following hypothesis is proposed;

**H**_**1**_: Academic stress among students in the period of COVID-19 significantly generates depression among students.

### Family Stress and Students' Depression Levels

Topuzoglu et al. (47) found that from 3 to 16.9% of the world's population suffers from depression. University students are more likely than the general population to experience depression. One-third of students (a subjective mean occurrence of 30.6%), as per Mirza et al.'s (48) study, report experiencing stress and depression, which indicates a 9% higher rate of depression among students than the general population (49). When depression sets in, a person's ability to lead an everyday, healthy life is severely impacted. Affecting students' social and family ties, academic performance, and physical wellbeing are just some of the possible outcomes of this problem. As a result, their abilities deteriorate, and they lose the desire to learn new things, which leads to subpar work and even university dropouts (50, 51). Depression is a significant risk factor for university students to commit suicide, so it is imperative to identify the factors that can cause students to become depressed.

In China, 75% of students who graduate from middle school are allowed to continue their education in a 4-year university. For every 10% increase in the number of students pursuing higher education, the rate of depression rises by 24–38% (52). University students are typically between the ages of 18 and 23, or in other words, in their late teens and early twenties. University students are referred to as “post-adolescents”. Risk factors for adolescent depression are numerous and complex, involving a wide range of personal and family characteristics and educational and social circumstances (16). Relationship building with one's family is one of the most important aspects of overcoming depression because it has a significant impact on the growth and development of one's children (53). According to Halonen et al. (54), adolescent depression is influenced by factors such as family bonding. Depressed teenagers are more likely to have strained relationships with their parents than their peers who are not depressed.

Both soft and hard risks can affect a family's health (55). Parents with little or no education are among the most challenging families to work with because of their weak family structure (economically). Depressed students are more likely to have several risk factors, including high-risk students (50). They have low self-esteem and cannot deal with emotional breakdowns, and students who come from families where they feel safe and secure (56). University students born into educated families, especially mothers with a college degree or higher, are less likely to suffer from depression than those born into families with little or no education. A second reason is that children born to college-educated mothers are less likely to suffer from depression than children of less-educated mothers (57).

While studying depression in teenagers, it is also essential to consider one's social circle (53). The traditional Pakistani culture emphasizes family ties, sensitive feelings, collectivism, and peace. Students who live in hostels or share a room with other students lose this family inspiration when they are adolescents. Depression is a real possibility if this process goes unchecked (58). It's not uncommon for Pakistani university students to worry about finding a job after graduation. They must maintain a high-grade point average (GPA) throughout their academic career if they hope to land an excellent job in the future. Aside from the pressures of school and work, they have to deal with a myriad of other issues that they must deal with on their own. In the pandemic period, students are also experiencing stress in their family life. It is claimed that this stress in the COVID-19 perspective creates depression among students. Thus, the following hypothesis is proposed;

**H2**: Family stress among students in the period of COVID-19 significantly generates depression among students.

### Students' Depression Levels and Students' Academic Performance

Many people who attend college represent the transition from adolescence to adulthood, generally regarded as the most stressful period of one's life (26). Students' mental health may be jeopardized if stress from exams and shifting social circles is added to the mix. One-third of students suffer from moderate to severe depression throughout their college careers (56). For depressed, attainable-focused environments (e.g., colleges and universities) can lead to lower grades and a lack of self-confidence because they believe the world is unfair and have no control over their destiny. They are unsure of their future path in life. As a result, students with low self-esteem are reluctant to take on challenging assignments and projects, harming their educational careers (59).

Mental and physical processes and benightedness can be found in symptoms such as poor sleep schedule, lack of concentration, and a state of remorse that can be seen in people with depression (60). However, despite many students suffering from depression and the flawed educational system, rare studies have examined the impact of depression on academic performance, particularly in COVID-19 in the emerging economies, specifically Pakistan. Students who are emotionally stable and financially secure are more likely to perform poorly on exams. Their academic career suffers significantly due to their low self-esteem and depression (59). Depressed students are more likely to skip classes, tests, and assignments. They are more likely to drop out of college than their non-depressed peers if they find their classes too complicated (26). Depressed students are prone to becoming ruthless, harming their academic performance and making them moody.

Anxiety and academic performance have been shown to have an even more ambiguous relationship than previously thought. Comprehensive studies have found that students' performance improves with increased anxiety (53). On the other hand, a few studies have found that anxiety does not appear to be correlated with lower academic performance (61). A higher level of anxiety can help students perform better in school. Even though there is a high incidence of depression among the students, their GPA is unaffected. This study is intended to find a more specific and straightforward answer to the shared relationship between students' depression levels and academic performance based on given differences in various research findings, particularly in the COVID-19 scenario. Based on the given arguments, the researcher formulates the following hypothesis:

**H3**: Depression among students during the COVID-19 period has a significant negative effect on their academic performance.

## Methodology

### Target Population and Sampling Procedure

Male and female students in higher education institutions focus on this study. Students from management sciences, engineering, and computer science departments participated and provided the researchers with their responses. The non-probability sampling technique has been used in this study, and students were given a survey to complete and asked to provide their thoughts on it on their own using a five-point Likert scale. Items for the study were taken from Maajida Aafreen et al.'s (43) study and were partially modified. The data were collected from February to May 2021. There were 721 questionnaires given out to the students, and 186 of those responses were useful. There were 118 female respondents, 65 male respondents, and one person who preferred not to disclose their gender.

## Data Analysis and Results

The structural equation modeling (SEM) method investigated the link between stress, depression, and academic performance. Before SEM, confirmatory factor analysis (CFA) was performed to confirm the relationship between the elements of the manifest factors and their measuring model. CFA ensures that the measurement model is legitimate and unidimensional. The Cronbach's alpha value was examined to ensure data reliability, which presented a 0.889 value confirming the reliability of data (minimum suggested value as is 0.6). Because of this, it can be concluded that this measurement model has a high degree of accuracy. In terms of psychological legitimacy, factor loading can determine the ideal loading for established items.

Similarly, the minimum value of the average variance extracted (AVE) for all results should be >0.5, which were found ideally fitted with the required value. Based on these, empirical tests were carried out to ensure that all constructs were distinct from one another. According to Fornell and Larcker (62), variance in results should be more significant than other constructs to make this claim. AVE square root values are correlated more strongly than different AVE values. To be safe, a correlation of no more than 0.9 was recommended by Hair et al. (63). Hair et al. (63) and Fornell and Larcker (62) suggested that the constructs have adequate discriminant validity by demonstrating that both conditions were met. The analysis of structural and measurement models indicated significant results. The authors examined different fit indices for structural and measurement models, such as chi-square to degree of freedom, normative fit index, goodness of fit index, comparative fit index, etc. According to Bagozzi and Yi (64) the value of fit indices should be higher than 0.9. Table 1 represents the list of fit indices with the suggested and obtained values of measurement and structural models.

**Table 1:** Evaluating the Structural and measurement models.

**The goodness of fit measures**	**CMIN/DF**	**NFI**	**GFI**	**AGFI**	**CFI**	**TLI**	**RMSEA**	**SRMR**
Recommended value	≤ 3^a^	≥0.9^b^	≥0.9^b^	≥0.9^b^	≥0.9^b^	≥0.9^b^	≤ 0.08^c^	≤ 0.08^d^
Measurement model	1.886	0.923	0.921	0.919	0.917	0.920	0.047	0.0557
Structural model	1.998	0.926	0.931	0.919	0.925	0.926	0.059	0.0673

a *Bagozzi and Yi (64)*.

b *Bentler and Bonett (65)*.

## Testing of Hypotheses and Discussion

This study examines the relationship between stress and depression and their impact on students' academic performance during the COVID-19 pandemic. Specifically, the authors focused on students in higher education institutions. They analyzed how the family and academic stress during the COVID-19 pandemic has emerged and how these elements impact students' academic performance. The hypotheses are examined using the SEM technique and are supported by structural parameters. According to the findings, students' academic stress promotes depression with a beta value of 0.298 and a *p*-value of 0.003. The results suggest that students' depression levels are positively impacted by prolonged academic stress, specifically in COVID-19. Thus, the first hypothesis, i.e., academic stress among students in the period of COVID-19 significantly generates depression among students, is accepted.

Family stress also appears to have a significant source of depression among students with 0.321 beta and 0.002 *p*-values, respectively. Thus, the second hypothesis, i.e., family stress among students in COVID-19, significantly generates depression, is also accepted. Similarly, the student's academic performance is negatively linked with the student's level of stress and depression. The structural analysis indicated that academic performance is negatively related to students' depression with a −0.332 beta value and 0.001 *p*-values. This means that the more depressed a student is, the more their academic performance will suffer. Thus, the third hypothesis, i.e., Depression among students during the COVID-19 period has a significant negative effect on their academic performance, is also accepted.

The results of this study provide university institutions with new opportunities to support students' psychological wellbeing and the conditions necessary to support it. There is a lack of support services for students' emotional wellbeing in higher education institutions, particularly in the COVID-19 pandemic. The psychological needs of these students are given little consideration, which is accelerating the stress and depression levels among students. Proper counseling, guidance from teachers, and family support can help alleviate stress and depression. Students should have access to stress and depression counseling services in their schools. Counselors have a responsibility to model and enforce good conduct and sound judgment in their students. Creating a positive and safe learning environment is the responsibility of school administrators. Teachers should also take responsibility for helping and guiding students who are depressed, as this will help them learn and perform better. The ability to rely on one's family for support is another critical factor in coping with stressful situations.

Limitations exist in the current research as well. Researchers collected information from university students in Pakistan. More research will be needed in the surrounding areas to understand better how stress and depression affect university students' academic performance. For this reason, researchers should expand their geographic scope to include other regions. The outcomes of large-scale studies may be inconsistent. Further research will be needed to determine the impact of anxiety and depression on students' academic performance in the future. Futhre studies are also recommended to include different control variables, such as age, gender, study discipline, etc., to examine whether these factors make any difference in the main result or not.

## Conclusion

The pandemic has created a social disorder in society, and all industries are affected by it. The academic sector is also primarily influenced by it. This is among the pioneer studies that examine the relationship between students' academic and family stress and their effect on their depression level, leading to academic performance, specifically in the period of the COVID-19 pandemic. It is found that education and family stress in the period of the COVID-19 pandemic has a hugely significant adverse effect on students' personalities. It is a substantial source of depression among students from their academic and family perspective and ultimately negatively affect their academic performance. All related stakeholders should take initiatives to counter this issue by counseling students to tackle this future generation's problem.

## Data Availability Statement

Publicly available datasets were analyzed in this study. This data can be found here: https://data.worldbank.org/.

## Author Contributions

ZJ and XJ: conceptualization, software, data curation, and writing—original draft preparation. RT: methodology, writing—reviewing, and editing. HD: visualization and investigation. All authors contributed to the article and approved the submitted version.

## Funding

This study was supported by Research Project of Shandong Provincial Department (Item Number Z2021290) and Education and Teaching Research Project of Shandong Province (Item Number 2021JXY053).

## Conflict of Interest

The authors declare that the research was conducted in the absence of any commercial or financial relationships that could be construed as a potential conflict of interest.

## Publisher's Note

All claims expressed in this article are solely those of the authors and do not necessarily represent those of their affiliated organizations, or those of the publisher, the editors and the reviewers. Any product that may be evaluated in this article, or claim that may be made by its manufacturer, is not guaranteed or endorsed by the publisher.
